# Impact of Influenza on Health-Related Quality of Life among Confirmed (H1N1)2009 Patients

**DOI:** 10.1371/journal.pone.0060477

**Published:** 2013-03-28

**Authors:** Malen Hollmann, Olatz Garin, Mariana Galante, Montserrat Ferrer, Angela Dominguez, Jordi Alonso

**Affiliations:** 1 Health Services Research Group, IMIM-Institut Hospital del Mar d'Investigacions Mèdiques, Barcelona, Spain; 2 Universitat Pompeu Fabra (UPF), Barcelona, Spain; 3 CIBER Epidemiología y Salud Pública (CIBERESP), Spain; 4 Universitat Autònoma de Barcelona (UAB), Barcelona, Spain; 5 Department of Public Health, Universitat de Barcelona (UB), Barcelona, Spain; University of North Carolina School of Medicine, United States of America

## Abstract

**Background:**

We aimed to assess the changes in health-related quality of life (HRQL) in patients with confirmed diagnosis of influenza (H1N1)2009, and to estimate the individual and societal loss of quality-adjusted life years (QALYs) caused by the pandemic.

**Methods and Results:**

Longitudinal study of patients recruited at major hospitals and primary care centers in Spain. Patients reported their HRQL (EQ-5D) during their influenza episode and seven days prior to it. A subsample was monitored to evaluate HRQL after recovery. HRQL loss was estimated as the difference between EQ-5D prior to the influenza episode and during it. Individual QALY loss (disutility multiplied by the duration of the influenza episode in days) for confirmed cases was calculated and used to estimate the societal loss in Spain (with the official estimations). A total of 432 inpatients and 563 outpatients were included, of whom 145 and 184, respectively, were followed up. Baseline mean HRQL loss was 0.58 (95% CI, 0.53–0.63) for inpatients and 0.43 (95% CI, 0.40–0.46) for outpatients. The majority of the 145 inpatients and 184 outpatients who were followed up regained initial HRQL levels, presenting a mean difference of 0.01 between the EQ-5D score prior to and after the influenza episode. Individual QALY losses for inpatients (0.031, 95% CI, 0.025–0.037) were higher than for outpatients (0.009, 95% CI, 0.007–0.011), while societal QALY losses were reversed: 94 years for inpatients and 6,778 years for outpatients. For fatal cases (an official number of 318), we estimated a QALY loss of 11,981.

**Conclusions:**

The influenza (H1N1)2009 pandemic had a significant but temporary impact on the HRQL of the majority of confirmed in- and outpatients. The societal impact of the influenza pandemic in Spain was estimated to be higher than other acute conditions. These results provide useful data for future cost-utility analyses.

## Introduction

Each year influenza epidemics result in substantial mortality and morbidity [1], [2]. It is estimated that an influenza epidemic affects 500 million people worldwide, causing 3 to 5 million severe cases of flu and 250−500 thousand deaths [3]. Occasionally, influenza outbreaks spread globally, giving rise to pandemics. In March 2009, a novel strain of influenza, (H1N1)2009, was detected in Mexico. By June 12, 2009, the infection had shown sustained human-to-human transmission across the world, leading the World Health Organization (WHO) to declare an influenza pandemic [4]. In Spain, for the 747,820 confirmed cases of (H1N1)2009 alone [5], [6], the economic healthcare burden was estimated to be €144,773,577 [7].

This information about influenza should be complemented with assessments that include disease burden or combined morbidity and mortality outcomes, such as disability-adjusted life years (DALYs) and quality-adjusted life years (QALYs). These outcomes have traditionally been used in the assessment of chronic conditions [8], [9], but little data is available for acute conditions like influenza [10]–[12]. Jan van Hoek et al. compared utility loss of confirmed influenza cases and influenza-like illness cases, and estimated an overall QALY loss during the (H1N1)2009 pandemic season in England of 22,267 QALYs [10].

In addition, and taking into account the high incidence of influenza [13]–[15] and its disabling capacity [10], [16], [17], it is also relevant to evaluate the individual loss of health-related quality of life (HRQL) in these patients. In fact, published evidence suggests an important reduction in HRQL during the disease, as well as a significant impact on the health system and the population [10], [12], [18]–[20]. Pradas et al. estimated a loss of HRQL of between 0.37 and 0.65 (on a scale from 0 to 1) due to a clinical influenza episode in outpatients during an epidemic wave in Spain [12]. However, no studies published to date have evaluated patients after recovery. Therefore, especially in a pandemic context, a more complete evaluation of the burden of influenza on HRQL is still lacking.

Such information should be relevant for decision making in health policy, most importantly, because it allows for the comparison: (a) between influenza and other health problems, (b) of the burden of influenza in different health systems, and (c) across time periods−between pandemics and epidemics or post-pandemic waves. It also highlights the need for measuring these indicators in continuous assessments. Furthermore, this information can be used to calculate the cost-utility rates of different preventive or treatment programs for influenza.

The aim of our study was to measure the impact of pandemic influenza (H1N1)2009 on HRQL in confirmed cases during and posterior to the influenza episode, and to estimate the associated individual and societal burden in Spain in terms of QALYs.

## Methods

### Study design and population

This was a multicenter, observational, longitudinal study of in- and outpatients with confirmed diagnoses of influenza (H1N1)2009, recruited for a larger government-commissioned project during the pandemic season in Spain (from its beginning to March 2010). Our research was a short-term follow-up study nested in a case-control study [7]. The case-control study aimed to investigate the risk factors of hospitalization for influenza (H1N1)2009 patients and the effectiveness of pharmaceutical and non-pharmaceutical interventions in its prevention [21]. Cases were defined as hospitalized patients with a confirmed diagnosis of influenza (H1N1)2009 admitted to one of the 36 participating hospitals in seven Spanish Autonomous Communities for influenza syndrome, respiratory failure, septic shock or multi-organ failure (“inpatient” for our study). For each case, four control patients, matched by age, time period of diagnosis and region of residence were recruited. One of these controls was in our interest, as it was a patient who had consulted a primary care center for flu syndrome and subsequently received a confirmed diagnosis of influenza (H1N1)2009, but did not require hospitalization (“outpatient” for our study). All influenza diagnoses were confirmed by real-time polymerase chain reaction (RT-PCR) as suggested by the WHO in the CDC protocol of real-time RT-PCR for Influenza A H1N1 [22].

For the results discussed here, in- and outpatients for whom baseline information on HRQL was incomplete and patients aged seven years old or younger were excluded (the HRQL instrument has not been validated for this age group [23]).

In addition, in pursuit of the secondary objective of this study, we contacted a subsample of patients (those for whom contact information was available) after recovery.

### Ethics statement

This study was approved by the Parc de Salut Mar ethics review board and was conducted in keeping with current Spanish law and the declaration of Helsinki. Written informed consent was obtained from all patients. In the case of children and adults with mental disabilities, written informed consent was obtained from the next of kin, caretakers, or guardians on behalf of these participants.

### Outcomes

The sociodemographic characteristics evaluated included sex, age, and social class based on the definitions of the Spanish Society of Epidemiology and dichotomized into class I to III (non-manual) and class IV and V (manual) and employment before influenza (among working-age adults, 16 to 64 years old). Information on underlying clinical conditions was gathered from clinical records, and included the presence of comorbidities such as respiratory or cardiovascular diseases, diabetes, obesity, immunosuppressive conditions, chronic renal failure, and rheumatologic or neuromuscular diseases. Women of childbearing age (15 to 50 years old) were asked whether they were pregnant at the time of the influenza episode. Data on many other variables were gathered but are not analyzed in this paper [24].

HRQL was measured with the EQ-5D questionnaire (Spanish adult and youth versions) [25], [26]. The EQ-5D is a standardized generic instrument that consists of two parts: the EQ-5D descriptive system (with the EQ-5D index) and the EQ visual analogue scale (EQ VAS) [25], [27], [28].

The EQ-5D descriptive system comprises five dimensions: mobility, self-care, usual activities, pain/discomfort and anxiety/depression. Each dimension has one specific question and three levels of response: 1 “no problems”, 2 “some problems” and 3 “severe problems”. The instrument therefore defines 243 distinct health states from all the possible combinations of dimensions and levels of severity (i.e., 3^5^). Considering the responses to the descriptive system, each health state is converted into a utility index by applying the general population preference values that, in the case of Spain, were obtained by means of the time trade-off method [25], [26]. The EQ-5D utility index ranges from 1 (best health status) to negative values (health states valued as worse than death, with a minimum of -0.6533), where 0 is equal to death. This utility index can be used to calculate QALYs. In addition, the EQ VAS consists of a self-rated health “thermometer” ranging from 0 “worst imaginable health state” to 100 “best imaginable health state”.

### Data collection

Patients were contacted at two different times: when they were recruited (baseline evaluation) and once they had recovered (follow-up evaluation). Recovery was defined as “clinical discharge from influenza”, determined through a set of questions included in the follow-up evaluation to ensure that the patient had overcome the influenza episode.

The baseline evaluation was completed by all the patients included in this study (hereafter referred to as the *complete sample*). The EQ-5D was administered with reference to two different recall periods: i) the day of hospital admission (inpatients) or of index medical visit (outpatients) (*HRQL during the influenza episode*), and ii) seven days prior to that event (*HRQL prior to the influenza episode*).

Patients included in the follow-up evaluation (hereafter *follow-up sample*) were asked about their clinical recovery from influenza, and if positive, they answered the EQ-5D with reference to that day (*HRQL posterior to the influenza episode*).

Because the outpatients were recruited as controls for the inpatients in the case-control study, and because the study started after the pandemic had begun, all of the outpatients and 80.9% of the inpatients were recruited retrospectively. The median time from hospital admission (inpatients) or index medical visit (outpatients) to recruitment was 145 days (IQR 91−187). The median time between the baseline interview and the follow-up evaluation was 123 days (IQR 64−176).

All the evaluations were carried out by trained interviewers and conducted face-to-face (14.4% of the baseline evaluations) or by telephone (85.6% of baseline evaluations and all follow-up evaluations). Patients' medical charts were also reviewed. In the case of children (8 to 17 years old) and adults with mental disabilities, a proxy was interviewed instead.

### Analysis strategy

All the analyses were stratified by type of patients (in- and outpatients). Descriptive analyses were conducted including distribution by sex, age, social class, employment, pregnancy and comorbidities. All comparisons of EQ-5D indexes were conducted with non-parametric tests (U-Mann Whitney, Kruskal Wallis and Wilcoxon tests) due to their highly skewed distributions [29].

#### HRQL impact

The response patterns to each EQ-5D dimension as well as to the EQ-VAS and EQ-5D indexes were described at each of the different time periods during the evolution of the influenza episode (before, during and after the influenza episode).

Additionally, two change indicators were used to describe the impact of influenza on HRQL: a) the proportion of patients that deteriorated in each EQ-5D dimension due to influenza, defining deterioration as the negative difference between the responses in the descriptive system during the influenza episode and prior to the influenza episode; and b) HRQL loss (disutilities−Δu) for each patient defined as the difference between the EQ-5D index prior to the influenza episode and the index during the influenza episode. The maximum EQ-5D index is 1.0 and the minimum is −0.65. Therefore, HRQL loss can range from 0 to 1.65.

Finally, the possible determinants of HRQL loss due to the influenza episode were explored by comparing HRQL loss differences among age groups and sociodemographic and clinical characteristics. A change in score of 0.07 was considered the minimal important difference [30].

#### QALY calculations

The quality-adjusted life year (QALY) is a disease measure which indicates the number of years of perfect health lost by a person or group due to mortality or disability. QALY calculations require data on individual utility loss and the time period for which that loss occurred (i.e., the duration of the influenza episode). In our study, the duration of the influenza episode was estimated as the number of days of absenteeism from work or school (the median was imputed when patients did not work or study). QALYs were estimated only in the follow-up sample, as that information was gathered during the follow-up evaluation.

Individual loss in terms of QALY (*ΔQ*) was calculated for each patient using the following formula,



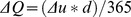
where *Δu* is the utility loss or disutility (HRQL loss) suffered by the patient, defined as the difference between the EQ-5D index prior to the influenza episode and the EQ-5D index during the influenza episode; and *d* is the duration in days of the influenza episode.

At the population level, the loss in QALYs was estimated using the mean QALY losses obtained at the individual level, and the number of confirmed cases in Spain during the season of the pandemic [5], [6]. QALY loss due to fatal cases was estimated imputing the mean population HRQL by age group at the time of death [29] and the corresponding life expectancy [31] to the actual number of confirmed deaths caused by influenza (H1N1)2009 in Spain [5].

## Results

**Figure 1:**
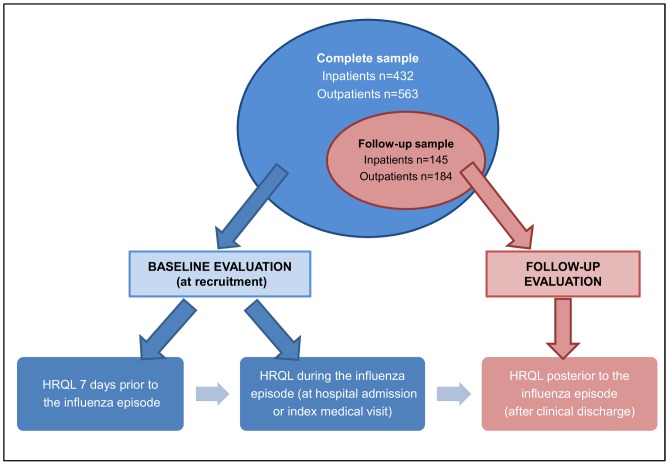
Baseline and follow-up samples and HRQL evaluation.

A total of 521 participants were excluded from the case-control sample (n = 1,516): 236 participants were younger than 8 years-old and 285 presented inconsistent or missing information on the EQ-5D. A total of 432 and 563 in- and outpatients met the criteria to be included in our analyses. From those, 145 inpatients and 184 outpatients were eventually followed up (**Figure 1**).

**Table 1** shows the characteristics of the patients in the complete and follow-up samples; and indicate the statistically significant differences between the follow-up sample and the remainder of the complete sample, for each type of patient (p <0.05).

**Table 1:** Sociodemographic and clinical characteristics of in- and outpatients with confirmed diagnoses of influenza H1N1(2009).

	Complete sample				Follow-up sample			
	Inpatients		Outpatients		Inpatients		Outpatients	
	n = 432		n = 563		n = 145		n = 184	
	n	%	n	%	n	%	n	%
Sociodemographic characteristics								
Sex (female)	234	54.2%	328	58.4%	76	52.4%	111	60.3%
Missing values			1	0.2%				
Age, mean (SD)	43.44 (19.08)		39.15 (16.13)		44.15 (19.20)		39.94 (15.43)	
8−18	64	14.8%	82	14.6%	20	13.8%	21	11.4%
19−34	75	17.4%	129	22.9%	23	15.9%	39	21.2%
35−49	121	28.0%	210	37.3%	43	29.7%	73	39.7%
50−64	115	26.6%	109	19.4%	39	26.9%	40	21.7%
>65	57	13.2%	33	5.9%	20	13.8%	11	6.0%
Social Class								
Non-manual	158	45.7%	262	54.5%	64	57.1%	119	73.9%*
Manual	188	54.3%	219	45.5%	48	42.9%	42	26.1%*
Missing values	86	19.9%	82	14.6%	31	21.7%	19	10.6%
Employment^1^	160/317	50.5%	350/470	74.5%	46/105	43.8%	104/156	66.7%
Missing values	5	1.6%	2	0.4%	5	4.5%	2	1.3%
Underlying clinical conditions								
Comorbidities	307	71.1%	210	37.3%	102	70.3%	94	51.1%*
Respiratory	193	44.7%	103	18.3%	52	35.9%*	40	21.7%
Obesity	79	18.3%	74	13.1%	37	25.5%	31	16.8%
Immunosuppressive condition	75	17.5%	38	7.1%	28	19.3%	18	9.8%*
Pregnancy^2^	36/129	27.9%	52/169	23.5%	9/41	22.0%	30/72	41.7%*
Missing values	3	2.3%	12	5.2%	3	6.5%	12	14.1%

1 Before the flu episode in adults of working age (16 to 64 years old); ^2^ in women of childbearing age (15 to 50 years old)

Missing values do not count toward the total percentages.

* Statistically significant differences between follow-up sample and the remainder of the complete sample, for each type of patient (p <0.05).

T student test was performed to compare mean age and a chi squared test was used for the rest of the comparisons.

Differences were found between in- and outpatients in both samples for all of the variables studied (p<0.05) except for sex. Among the inpatients, more than fifty percent were women, their mean age was around 43 years of age, and approximately 40% were 50 years old or above. In the baseline group, 54.3% were classified in the lower social class group (this percentage decreased in the follow-up samples to 42.9%). Only half of the inpatients of working age were actually working before the influenza episode. More than 70% of the inpatient group had at least one comorbid condition; and between 2.3% and 6.5% of the women of childbearing age were pregnant in the baseline and follow-up samples, respectively.

Nearly 60% of the outpatient group were women, had a mean age of about 40 and 25% were 50 years old or older. 45.5% and 26.1% were considered in social class III or IV in the complete and follow-up samples, respectively. Around 75% of the working-age outpatients were employed before the influenza episode. At least one comorbid condition was recorded in 37.3% of the outpatients in the complete sample, and in 51.1% of the follow-up sample.

As shown in **table 2**, the EQ-5D index prior to the influenza episode was 0.81 (95% CI, 0.78–0.84) on average for inpatients, and 0.93 (95% CI, 0.91–0.96) for outpatients. In both groups the index changed significantly (p<0.001) during the influenza episode, decreasing to 0.23 (95% CI, 0.10–0.28) and 0.50 (95% CI, 0.46–0.53) respectively. Previous to the influenza episode, the majority of inpatients reported “no problems” (ranging from 64.4% for pain/discomfort to 85.2% for self-care) in all dimensions, but the number of “no problems” reports decreased during the influenza episode (ranging from 14.6% in pain/discomfort to 56.3% in self-care). Similar trends were found among outpatients.

**Table 2:** HRQL evolution of in- and outpatients with confirmed diagnoses of influenza H1N1(2009).

	Prior to the influenza episode			During the influenza episode		
Inpatients (n = 432)						
	Mean	Median	95% CI	Mean	Median	95% CI
EQ-5D index	0.81	1.00	0.78 - 0.84	0.23	0.22	0.18 - 0.28
EQ VAS^1^	66.2	70	63.7 - 68.7	55.0	60	52.2 - 57.9
Descriptive EQ-5D system (%)	No problems	Some problems	Severe Problems	No problems	Some problems	Severe Problems
Mobility	80.1	15.3	4.6	41.2	29.4	29.4
Self-care	85.2	10.6	4.2	56.3	22.5	21.3
Usual activities	74.5	18.5	6.9	33.8	24.5	41.7
Pain/discomfort	64.4	29.9	5.8	14.6	33.6	51.9
Anxiety/depression	81.5	16.9	1.6	50.7	50.7	16.9
Outpatients (n = 563)						
	Mean	Median	95% CI	Mean	Median	95% CI
EQ-5D index	0.93	1.00	0.91–0.96	0.50	0.65	0.46–0.53
EQ VAS^2^	78.7	85	76.8–80.5	62.8	70	60.5–65.1
Descriptive EQ-5D system (%)	No problems	Some problems	Severe Problems	No problems	Some problems	Severe Problems
Mobility	93.3	5.9	0.9	68.4	21.1	10.5
Self-care	96.4	2.7	0.9	79.4	15.8	4.8
Usual activities	91.5	7.1	1.4	44.4	30.9	24.7
Pain/discomfort	81.2	16.9	2.0	12.8	49.9	37.3
Anxiety/depression	92.0	6.0	2.0	71.6	19.4	9.1

Comparisons between EQ-5D indexes and VAS reported prior to the flu episode and during it were conducted with the Wilcoxon test.

1 Missing values: 3 for prior to the influenza episode and 3 for during the influenza episode.

2 Missing values: 6 for prior to the influenza episode and 5 for during the influenza episode.

**Figure 2:**
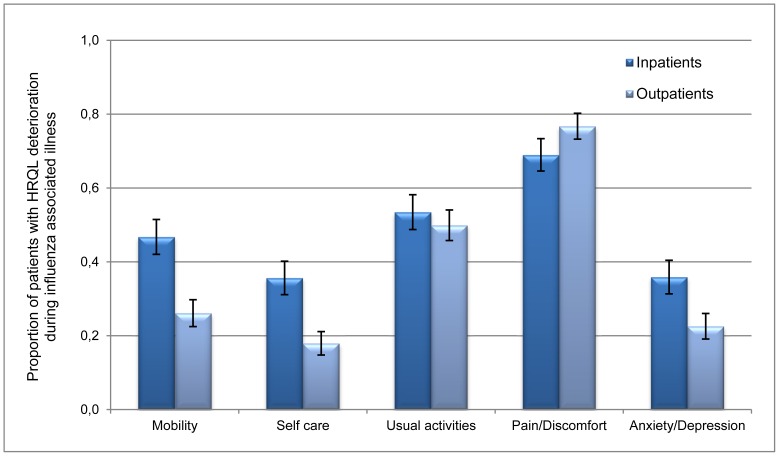
Proportion of patients with confirmed diagnoses of influenza H1N1(2009) who presented a deterioration in HRQL, shown by EQ-5D dimensions.

**Figure 2** shows the proportion of patients with deterioration per dimension. Except for pain/discomfort, the degree of deterioration in all the dimensions was less for outpatients than for inpatients (p<0.001). Pain/discomfort and usual activities were the most affected dimensions, for both in- and outpatients (ranging from 0.50 to 0.77).

**Figure 3:**
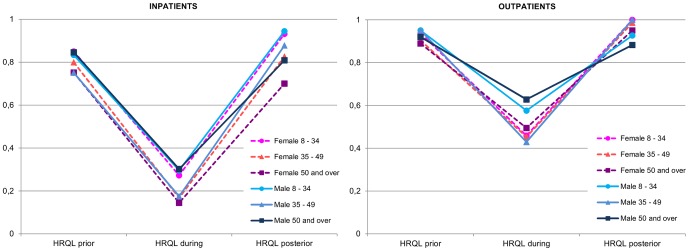
HRQL evolution of in- and outpatients with pandemic influenza H1N1(2009), stratified by sex and age group. HRQL prior to and during the flu episode indexes correspond to the entire sample (inpatients: 432 and outpatients: 563) and HRQL posterior to the flu episode indexes correspond only to the follow-up sample (inpatients: 145 and outpatients: 184).

**Figure 3** describes the in- and outpatients' EQ-5D index changes over time, by sex and age group. Differences between EQ-5D prior to and during the influenza episode were evaluated within the complete sample, while differences between EQ-5D prior to and posterior to the influenza episode were only assessed within the follow-up sample. The EQ-5D index decreased significantly during the influenza episode in all subgroups (p<0.001). Overall, in- and outpatients showed full recovery posterior to the influenza episode (0.83 vs. 0.84 for inpatients, p-value 0.97, and 0.95 vs. 0.96 for outpatients, p-value: 0.102, data not shown). Only in the male over-50 outpatient group (n = 57) was the EQ-5D index significantly lower after the influenza episode (0.96 vs. 0.87, p-value 0.017). EQ-5D scores were higher after the influenza episode than before it in two outpatient groups: 8−34 year old females (n = 116) and 35−49 year old males (n = 82) (p<0.05).

**Table 3** shows the change in the EQ-5D index prior to and during the influenza episode (HRQL loss) for all in- and outpatients, stratified by sex. Mean HRQL loss due to influenza was 0.58 (95% CI, 0.53–0.63) for inpatients, and 0.43 (95% CI, 0.40–0.46) for outpatients. Overall, the loss presented by inpatients was greater than that of outpatients (p<0.001, data not shown).

**Table 3:** HRQL loss according to sociodemographic and clinical variables, stratified by sex.

	Inpatients						Outpatients					
	All		Female		Male		All		Female		Male	
	n = 432		n = 234		n = 198		n = 563		n = 328		n = 234	
	Mean	95% CI	Mean	95% CI	Mean	95% CI	Mean	95% CI	Mean	95% CI	Mean	95% CI
Whole sample	0.58	0.53 – 0.63	0.60	0.54 – 0.67	0.55	0.48 – 0.62	0.43	0.40 – 0.46	0.45	0.40 – 0.49	0.41	0.35 – 0.46
Age groups												
8−18	0.54	0.41 – 0.67	0.57	0.40 – 0.75	0.50	0.30 – 0.70	0.41	0.32 – 0.51	0.55	0.37 – 0.73	0.34	0.23 – 0.45
19−34	0.58	0.48 – 0.69	0.58	0.44 – 0.72	0.59	0.40 – 0.77	0.44	0.37 – 0.52	0.45	0.37 - 0.54	0.42	0.28 – 0.56
35−49	0.61	0.52 – 0.70	0.63	0.52 – 0.75	0.58	0.44 – 0.71	0.48	0.42 – 0.54	0.45	0.38 – 0.52	0.52	0.43 – 0.61
50−64	0.58	0.49 – 0.67	0.61	0.48 – 0.74	0.55	0.41 – 0.68	0.36	0.29 – 0.43	0.41	0.32 – 0.50	0.30	0.19 – 0.40
>65	0.56	0.43 – 0.69	0.59	0.37 – 0.81	0.54	0.37 – 0.71	0.32	0.20 – 0.45	0.36	0.18 – 0.53	0.27*	0.08 – 0.46
Social Class												
Non–manual	0.53	0.46 – 0.61	0.55	0.46 – 0.64	0.51	0.38 - 0.63	0.39	0.35 – 0.44	0.40	0.35 – 0.46	0.38	0.30 – 0.46
Manual	0.62	0.55 – 0.69	0.64	0.59 - 0.75	0.60	0.50 – 0.70	0.45	0.39 – 0.50	0.47	0.39 – 0.54	0.43	0.34 – 0.51
Employment^1^												
No	0.55	0.48 – 0.63	0.57	0.47 – 0.67	0.53	0.42 – 0.65	0.44	0.37 – 0.52	0.48	0.39 – 0.58	0.36†	0.22 – 0.50
Yes	0.62	0.54 – 0.70	0.64	0.54 – 0.75	0.59	0.47 – 0.71	0.45	0.41 – 0.49	0.43	0.38 – 0.48	0.48*	0.41 – 0.55
Comorbidities												
No	0.53	0.44 – 0.61	0.58	0.46 – 0.69	0.45	0.32 – 0.58	0.44	0.39 – 0.48	0.44	0.39 – 0.49	0.43	0.36 – 0.50
Yes (Any)	0.60	0.55 – 0.66	0.62	0.54 – 0.69	0.58	0.50 – 0.66	0.42	0.36 – 0.47	0.46	0.38 – 0.54	0.36	0.28 – 0.44
Respiratory	0.62	0.55 – 0.68	0.64	0.54 – 0.74	0.59	0.50 – 0.68	0.41	0.32 – 0.50	0.47	0.35 – 0.59	0.32	0.20 – 0.44
Pregnancy^2^												
No	--	--	0.64	0.53 – 0.74	--	--	--	--	0.48	0.42 – 0.54	--	--
Yes	--	--	0.54	0.40 – 0.68	--	--	--	--	0.43	0.31 – 0.54	--	--

1 Prior to the flu episode in adults of working age (16 to 64 years old); ^2^in women of childbearing age (15 to 50 years old.)

† Statistically significant differences when comparing the change in EQ-5D in females and males (p <0.05).

* Statistically significant differences when comparing the EQ-5D change among specific characteristics in each group or sex group (p <0.05).

A U-Mann Whitney test or Kruskal Wallis test were performed to compare means HRQL losses.

Although there were no statistically significant differences found between the HRQL loss of in- and outpatients by groups, a change in score of 0.07 was considered an important difference [30]. Among inpatients, patients in the manual social class and employed patients experienced a greater degree of deterioration (0.62 vs. 0.53 and 0.62 vs. 0.55, respectively). The same differences in HRQL loss were observed in inpatients with any comorbidity (0.60 vs. 0.53), especially for those with a respiratory problem (0.62). For pregnant women, the impact on HRQL was lower than that for non-pregnant women of the same age group (0.54 vs. 0.64).

Regarding HRQL loss comparisons between sexes, statistically significant differences (p<0.05) were only found among unemployed outpatients (0.48 female vs. 0.36 male). Changes in the EQ-5D index for in- and outpatient females did not differ by age group, social class, employment status, comorbidities or pregnancy. For outpatient males, the loss differed significantly according to age (from 0.27 for elderly subjects to 0.52 for middle-aged subjects, p-value: 0.014) and employment status (0.48 employed vs. 0.36 unemployed, p-value: 0.047).

The loss in HRQL for the inpatient follow-up sample was 0.61 (95% CI, 0.53–0.70) and 0.42 (95% CI, 0.36–0.48) for the outpatient follow-up sample. The median duration of the influenza episode was 21 days (IQR 10−36) for inpatients while it was 7 days (IQR 5.5−10) for outpatients. The resulting individual QALY loss was 0.031 (95% CI, 0.025–0.037) for inpatients and 0.009 for outpatients (95% CI, 0.007–0.011). Considering the confirmed 3,025 inpatient and 744,795 outpatient (H1N1)2009 cases [5], [6] in Spain, the overall burden of the pandemic influenza season was about 94 QALYs for inpatients and 6,777 for outpatients (**Table 4**). Case fatality data (318 deaths) was estimated to result in a loss of 11,981 QALYs (**Table 5**).

**Table 4:** QALYs losses due to influenza (H1N1)2009 in Spain at the individual and population level, stratified by in- and outpatients.

	Inpatients	Outpatients
	(n = 145)	(n = 184)
Utility loss mean (95% CI)	0.61 (0.53 – 0.70)	0.42 (0.36 – 0.48)
Duration in days of influenza episode. Median (IQR)	21 (10 – 36)	7 (5.5 – 10)
Individual QALYs mean (95% CI)	0.031 (0.025 – 0.037)	0.009 (0.007 – 0.011)
Cases^1^	3,025	744,795
Total QALYs loss	94.08	6,777.63

1 Data taken from Santa-Olalla study for inpatient confirmed cases [6] and from Larrauri study for outpatient confirmed cases [5].

**Table 5:** Estimated QALY loss due to case fatality data caused by influenza (H1N1)2009 in Spain.

Age	Deaths^1^ [5]	Life expectancy^1^ [31]	EQ-5D score^1,2^ [29]	Δu	ΔQ individual	Total QALYs loss
(years)	(N)	(years)	Mean (SE)			
0–4	16	81.83	0.94 (0.004)	0.94	76.92	1,230.72
5–14	13	75.39	0.94 (0.004)	0.94	70.87	921.27
15–64	223	45.85	0.90 (0.006)	0.90	41.27	9,202.10
>64	66	13.37	0.71 (0.020)	0.71	9.49	626.52
Total QALYs loss due to fatality cases in Spain						11,980.60

1 Data from Larrauri study [5], SISalut website [31] and Cunillera study [29].

2 The EQ-5D score for the corresponding age group at the time of death was imputed for the remaining life expectancy.

## Discussion

Influenza (H1N1)2009 caused a great and significant impact on both in- and outpatients with a HRQL deterioration of 0.58 and 0.43, respectively (the maximum possible EQ-5D score is 1.0 and the minimum is -0.65, corresponding to a maximum value change of 1.65.). Nevertheless, the vast majority of individuals recovered their levels of HRQL after clinical discharge. Taking into account its high incidence and the magnitude of its impact, influenza (H1N1)2009 caused a considerable loss in QALYs among the Spanish population affected by the pandemic: more than 6,870 QALYs among confirmed cases alone, and more than 11,980 among fatal cases.

The main strengths of our study included a) a multicenter and prospective design that allowed for the full, comprehensive assessment of the impact of influenza episodes b) a follow-up evaluation to determine the extent to which patients completely recovered their HRQL after the episode and to estimate the duration of the disease; and c) the use of a well-known HRQL instrument such as the EQ-5D questionnaire.

When interpreting the results of our study, some limitations should be considered. First, a selection bias may have occurred. Patients who died during the influenza episode were excluded from the study. Although the national health monitoring system recorded these deaths, we have no information regarding their HRQL before the influenza infection. Therefore, the HRQL of the corresponding age group in the general population was used to calculate the QALY loss due to fatal cases. This imputation may have overestimated our results. First, because we have not considered the HRQL index declination over time with age, and second, because these patients very likely had comorbidities and would have presented lower HRQLs than the general population, a situation that would have also deteriorated with age. Moreover, only a subsample of patients was followed up after recovery, and outpatients were selected as matched controls for inpatients, which may limit the ability to draw generalizations based on our results. Nevertheless, the follow-up samples and the remainder of the complete samples were shown to be comparable; the outpatients' HRQL prior to the influenza episode was similar to that of the general population in Catalonia, 0.93 vs. 0.88 (IQR: 0.87–1.00)[29]; and the temporal distribution of cases was representative of the pandemic wave in Spain (data not shown)[5]. Second, HRQL information was gathered retrospectively in the majority of cases and we believe that the mass media coverage of the flu pandemic might have affected the recall bias in the patients with regard to their health status[32]; a possible response shift may be responsible for the slightly improved reports of quality of life after recovery in some groups [33]. Responses obtained by a proxy might also be biased, but the discriminative ability shown by the patients at the different stages of this study and the extensive use of the EQ-5D proxy version support the response validity [34]–[37]. Third, for the QALY loss results it should be noted that: a) considering the HRQL/utility reported for one day out of the entire influenza episode might overestimate the loss of QALY per patient (as the patient most likely remembers the day that he or she considers the worst); b) the duration of the influenza episode was estimated as the number of days of absenteeism from work or school; and c) the weights applied to the children are adult-derived weights, given that there are currently no children-specific weights available. Finally, the sample size was insufficient to allow us to identify the clear determinants of HRQL deterioration or the heterogeneity upon recovery.

### HRQL deterioration

Several differences were identified between in- and outpatient characteristics (age, social class, employment, comorbidities and pregnancy). Some of these differences might be a direct consequence of the characteristics of the pandemic (e.g. pregnancy and age) [5], [38]–[41], but others may be common to any seasonal epidemic. For instance, as is typical with seasonal influenza, most inpatients were people with a high frequency of comorbidities (70.3%) [42]–[44]. In addition, the HRQL of inpatients prior to the influenza episode was poorer than that of the general population in Catalonia, 0.81 vs. 0.88 (IQR: 0.87–1.00)[29]. As expected, HRQL loss due to influenza infection was higher among inpatients (0.58 vs. 0.43 for outpatients), although their HRQL index prior to the influenza episode was lower than that of outpatients. Among inpatients, lower social class and being employed seem to be associated with a higher degree of deterioration. As expected, the presence of any comorbidity, especially a respiratory comorbidity, may also be a related factor for a greater HRQL loss due to influenza. Unexpectedly, pregnancy seems to be associated with a lesser degree of deterioration in the HRQL of women. Santa-Olalla et al. reported that the prevalence of pregnant inpatients during the pandemic season in Spain was higher than the prevalence of female inpatients of childbearing age [6]. As pregnancy had initially been reported as a possible risk factor for complications among people with influenza [41], [45], the lower impact observed in our study might be due to the hospitalization of patients whose cases were relatively mild.

Although there were no statistically significant differences in the HRQL loss between women and men (within in- or outpatients), in most stratified gender comparisons HRQL loss tended to be higher among women and frequently above the minimal important difference (0.07) [30]. The possible differential impact of influenza among women deserves further research.

### The importance of evaluating recovery

Most patients reported the same HRQL before and after the influenza episode and some groups even presented better HRQL scores after the influenza episode than prior to it. These results might be slightly affected by the short seven-day timeframe for the evaluation prior to the influenza episode, and by the possible bias mentioned earlier.

These findings regarding the complete recovery of most patients show that influenza is a temporary disorder with no mid-term consequences for the majority of the population and they allowed the calculation of QALYs without doubts regarding possible un-measured retained impairments after the influenza episode.

### Loss in quality-adjusted life years

In support of the above mentioned similarity between our results (regarding HRQL deterioration and the duration of the episode) and those found in previous studies, the mean loss of QALY per patient (individual burden) from our data was similar to that reported by previous European studies [10], [12]. However, some methodological differences need to be taken into consideration. Pradas et al. studied outpatients in Spain during an epidemic wave, while the van Hoek et al. study included in- and outpatients during the pandemic season in England. Although the duration of the influenza episode has been calculated by means of different strategies in some studies [10] and extrapolated from a literature review in others [12], the results are similar when compared with the outpatient group, from 7.3 to 10.5 days. Despite the differing characteristics of these works, our individual QALY loss data (0.031 for inpatients and 0.009 for outpatients) is consistent with that reported in both studies (0.014 and 0.008 by Pradas and van Hoek, respectively).

At the population level, our results showed a considerable overall burden caused by the influenza (H1N1)2009 pandemic in Spain, with more than 6,870 QALYs lost solely among confirmed cases. If we considered all of the 1,414,000 clinical cases estimated in the Spanish pandemic, the loss of QALYs at the population level would rise to 12,820, or to 24,801 if the estimated loss due to death is also considered. In any case, this is lower than the burden estimated in Spain due to chronic processes like diabetes mellitus (98,700 DALYs in 2006, bear in mind that DALY and QALY results might not be directly comparable) [46] or mental disorders, with an annual QALY loss of 1,831 per 100,000 patients [47]. Moreover, when compared with other similar acute pathologies, like otitis media or appendicitis, which have been associated with an annual burden of 1,004 and 3,012 DALYs respectively [46], the relevance of the burden of influenza becomes clear.

### Conclusion

Our study confirmed the significant impact of the influenza (H1N1)2009 pandemic on patients' HRQL as well as its recovery after the influenza episode. Additionally, the pandemic has been found to have caused a considerable loss in QALYs, comparable with the burden of some chronic diseases and higher than that of other acute-infectious diseases (even though only confirmed influenza infections were considered). These results provide original information that can be applied in cost-utility analyses, help in decision-making processes in health management and provide support to the development of preventive public health policies.
